# Exploring the Diversity and Aromatic Hydrocarbon Degrading Potential of Epiphytic Fungi on Hornbeams from Chronically Polluted Areas

**DOI:** 10.3390/jof7110972

**Published:** 2021-11-16

**Authors:** Valeria Imperato, Miguel Portillo-Estrada, Anabel Saran, Anneleen Thoonen, Łukasz Kowalkowski, Stanislaw W. Gawronski, Francois Rineau, Jaco Vangronsveld, Sofie Thijs

**Affiliations:** 1 Department of Biology, Centre for Environmental Sciences, Hasselt University, BE3590 Diepenbeek, Belgium; thoonen.anneleen@gmail.com (A.T.); lukaszkowalkowski.biotech@gmail.com (Ł.K.); francois.rineau@uhasselt.be (F.R.); jaco.vangronsveld@uhasselt.be (J.V.); sofie.thijs@uhasselt.be (S.T.); 2 Plants and Ecosystems (PLECO), Department of Biology, University of Antwerp, BE2610 Wilrijk, Belgium; miguel.portilloestrada@uantwerpen.be; 3 AIC-CONICET, Scientific Research Agency, Santa Rosa 6360, La Pampa, Argentina; saran.anabel@gmail.com; 4 Faculty of Horticulture, Biotechnology and Landscape Architecture, Warsaw University of Life Sciences, 02-787 Warsaw, Poland; stanislaw.gawronski@gmail.com; 5 Department of Plant Physiology and Biophysics, Faculty of Biology and Biotechnology, Maria Curie-Skłodowska University, 20-400 Lublin, Poland

**Keywords:** air pollution, phyllosphere, phylloremediation, fungi

## Abstract

Plants can ‘catch’ and mitigate airborne pollutants and are assisted by fungi inhabiting their leaves. The structure and function of the fungal communities inhabiting the phyllosphere of hornbeam trees growing in two chronically polluted areas, the oilfield of Bóbrka and the city center of Warsaw, were compared to the ones growing in one nature reserve, the Białowieża National Park. Fungi were isolated and characterized both phylogenetically and functionally for their potential role in air pollution mitigation. Both culture-dependent (e.g., enzyme assays and tolerance tests) and culture-independent methods (e.g., ITS and shotgun sequencings) were used. Furthermore, the degradation potential of the fungi was assessed by gas chromatography mass spectrometry (GC-MS). Shotgun sequencing showed that the phyllosphere fungal communities were dominated by fungi belonging to the phylum Ascomycota. *Aureobasidium* was the only genus detected at the three locations with a relative abundance ≥1.0%. Among the cultivated epiphytic fungi from Bóbrka, *Fusarium sporotrichioides* AT11, *Phoma herbarum* AT15, and *Lophiostoma* sp. AT37 showed in vitro aromatic hydrocarbon degradation potential with laccase activities of 1.24, 3.62, and 7.2 μU L^−1^, respectively, and peroxidase enzymes with activities of 3.46, 2.28, and 7.49 μU L^−1^, respectively. Furthermore, *Fusarium sporotrichioides* AT11 and *Phoma herbarum* AT15 tolerated exposure to airborne naphthalene and benzene. *Lophiostoma* sp. AT37 was the most tolerant to exposure to these pollutants, in line with being the best potential aromatic hydrocarbon degrader isolated in this study.

## 1. Introduction

Outdoor air pollution is an invisible killer, causing 4.2 million human deaths in the world each year [1]. New data reveal that 9 out of 10 people breathe air containing high levels of pollutants as particulate and volatile organic compounds (VOCs), including mono- and polycyclic aromatic hydrocarbons (MAHs and PAHs). 

Plants are known to ‘catch’ and mitigate airborne pollutants. Especially plant leaves can adsorb pollutants passively, and subsequently, pollutants can be up taken by plant cells and/or chemically transformed [2,3]. Because of the significant surface area they span on Earth, which is estimated as a global leaf area of 508,630,100 km^2^, leaves can have a substantial impact as a method for filtering air [4]. Plant leaves are not only a passive surface area for air filtration, they are also the habitat for thousands microbial cells, including fungi and bacteria [5]. The phyllosphere can be defined as the aerial parts of living plants, which includes leaves, stems, buds, flowers, and fruits that provide a habitat for microorganisms [4]. Studies on phyllosphere microbiology have been mainly focusing on leaves, which are the plant structures with the highest global area. The leaf phyllosphere provides nutrients and a shelter for a large range of highly diverse microorganisms. Nevertheless, differently from other environmental niches, the conditions in the leaf phyllosphere can be considered as extreme due to the exposure to UV radiation and extreme temperatures [6,7]. Season also play an important role in structuring the phyllosphere microbial communities. According to Gomes et al. [8], seasoning was one of the major drivers in shaping the epiphytic community, whereas wind speed, rainfall, and temperature were the major drivers shaping the endophytic community. 

The most dominant colonizers of the phyllosphere are bacteria with 10^6^ to 10^7^ cells cm^−2^ of leaf. Less is known about numbers of phyllosphere fungi [7]. Biomass, more than cell number, can probably be a more adequate nonbiased parameter for comparing the dominance of fungi or bacteria on leaves. Biomass is the total weight of organism in a given area, which is directly related to the microbial size [9]. A few studies have evaluated phyllosphere biomasses associated to plants, as in the case of Davey et al. [10], who investigated fungal biomass associated with the phyllosphere of bryophytes and vascular plants, and Jia et al. [11], who investigated fungal communities during plant development in a copper tailings dam. 

Phyllosphere microorganisms, since they are continuously in contact with air and consequently with the airborne aromatic hydrocarbons, can cooperate in filtering and degrading air pollutants and consequently, improve air quality [12]. For instance, fungal communities of ornamental plants on roadsides in Sri Lanka adapted to pollution conditions by shaping into communities able to degrade aromatic hydrocarbons [13]. Fungi possess a higher capability to degrade aromatic hydrocarbons compared with other organisms, mainly because of the secretion of extracellular enzymes as lignin peroxidases (LiP), manganese peroxidases (MnP), and laccases (ligninolytic fungi), whereas others instead produce cytochrome P450 monooxygenase-like enzymes (non-ligninolytic fungi) [14,15]. 

In light of the above, phylloremediation, which is based on the synergistic action of plants and their associated microorganisms to degrade airborne pollutants, is a promising green technology to tackle the air pollution issue. Engineers and plant scientists are focusing on harnessing the potential of plants and their associated microorganisms to mitigate air pollution [2,16,17]. Until now, our knowledge about the interactions of microorganisms with plant leaves has been limited. This shortage of knowledge is a main obstacle to further investments and installations of urban green with the purpose for phylloremediation. 

The overall aim of this study is to get a better insight in the community structure and function of epiphytic fungi associated with common hornbeam, via culture-dependent and -independent methods, to further pave the way for future remediation applications. For this purpose, we specified the following objectives: (I) to genotypically characterize the fungal communities on leaves of hornbeam, (II) to culture some representatives and identify them by Sanger sequencing of the ITS and TEF regions, (III) to test them for laccase and peroxidase enzyme activity, and (IV) to perform in vitro tests for airborne pollutant degradation.

## 2. Material and Methods

### 2.1. Sampling of Hornbeam Leaves from Three Differently Polluted Areas in Poland

To investigate the fungal communities living on the leaves of hornbeam trees and to assess their potential for degradation of aromatic hydrocarbons (AH), three sites in Poland were selected for sampling. Białowieża National Park is Europe’s last temperate primeval forest with a minimal anthropogenic disturbance (52.7229° N, 23.6556° E). This forest is a UNESCO world heritage site and an EU Natura 2000 special area of conservation [18]. Bóbrka oil industry museum is a unique site where the environment naturally was in contact with crude noncombusted oil since the last glacial period (52.8167° N, 23.9264° E). Bóbrka has the world’s oldest oil wells with still a regular output of a barrel per well every day [19]. Warsaw city center (52.2297° N, 21.0122° E) air pollution mainly originates from combusted exhaust-related organic volatiles, industry, and other human activities. These sampling locations are far from each other, from a minimum of around 230 km (Warsaw and Białowieża) to a maximum of around 460 km (Bóbrka and Białowieża), and were chosen based on different levels of air pollution. More details about the locations and sampling have been previously described [20].

For each site, five locations were sampled (May 2016), and at each location, four trees and five leaves per tree were collected for microbiome analyses (4 × 5 = 20 biological replicates per location) at a height of 1.5 to 2 m. Leaves were collected with ethanol-sterilized metal scissors and tweezers and stored into sterile 50 mL Falcon tubes previously filled with phosphate buffer containing (per liter): 11.95 g NaH_2_PO_4_ 2H_2_O, 16.5 g Na_2_HPO_4_ 7H_2_O, and 100 μL Tween 80. Samples were transported ice-cooled to the laboratory for further processing.

### 2.2. Isolation of Biomass from Hornbeam Phyllosphere 

In order to isolate epiphytic fungi, 50 mL tubes containing hornbeam leaves in 10 mM phosphate buffer were inverted by hand for 10 s, sonicated for 3 min at 160 W using a Bransonic^®^ ultrasonic cleaner 2510E-MT (Branson Ultrasonics Corp., Danbury, CT, USA), vortexed for 1 min, and lastly, shaken on an orbital shaker for 15 min at 240 rpm. Leaves were removed from the tubes, and the phosphate buffer was centrifuged (4000 RCF × 15 min) to pellet epiphytic microbial cells [21]. The supernatant was discarded, and the pellet was resuspended in the remaining 3 mL of phosphate buffer to allow the further steps: (i) total genomic DNA isolation and analyses by ARISA and shotgun metagenomic sequencing and (ii) culturing epiphytic fungi.

### 2.3. Total gDNA Extraction from Leaf Wash Suspensions 

One milliliter of the cell suspensions was transferred into sterile 2 mL tubes. Cells were pelleted by centrifugation (10,000 RCF × 15 min), supernatant was discarded, and cells were lysed by homogenization into 180 μL of lysis buffer (1 × Tris-EDTA buffer pH = 8, 1.2% Triton X-100 and 20 mg mL^−1^ lysozyme in Rnase-free water) and incubated at 37 °C for 30 min. Subsequently, 25 μL of proteinase K and 200 μL of AW buffer from the Qiagen Blood & Tissue kit (Qiagen, Venlo, The Netherlands) were added, followed by an incubation at 56 °C for 30 min. The gDNA isolation protocol of the Qiagen kit was used. The DNA purity (260/280 nm and 260/230 nm) was assessed with a NanoDrop ND-1000 spectrophotometer (Isogen Life Science, The Netherlands), the DNA concentration was quantified using the QuantiFluor^®^ dsDNA System (Promega, The Netherlands), and its integrity was assessed. DNA gel-loading dye (6X) by Thermo Fisher (US) was used before loading the samples in an agarose gel 1.5% (120 V; 45 min). Finally, gDNA samples were stored at −20 °C for further analyses.

### 2.4. Community Fingerprint Analyses

Automated ribosomal intergenic spacer analysis [22,23] was carried out to investigate the composition of hornbeam phyllospheric communities and to compare the hornbeam phyllospheres from (i) the crude-oil-polluted area of Bóbrka, (ii) the nature reserve of Białowieża, and (iii) the city center of Warsaw. The fungal intergenic spacer region ITS1-5.8S-ITS2, i.e., the region between the fungal 18S rRNA gene and the 28S rRNA gene, was amplified using the primer pair 2234C (5′-GTTTCCGTAGGTGAACCTGC-3′) and 3126T (5′-ATATGCTTAAGTTCAGCGGGT-3′) [23,24]. Firstly, for the PCR amplification, samples were prepared as follow: 5 μL 10X hifi PCR buffer (Roche, Basel, Switzerland), 2 μL 50 mM MgSO_4_, 1 μL dNTP mix, 0.1 μL of each primer (0.1 mM), 38.8 μL RNase-free water, and 0.2 μL Platinum^®^ Taq high fidelity (Invitrogen, Waltham, MA, USA) for a total of 50 μL reaction mixture. To each reaction, 1 μL of DNA (15–40 ng μL^−1^) extracted from the different leaf washes was added. PCR conditions consisted of an initial denaturation at 95 °C for 3 min, followed by 30 cycles of 95 °C for 1 min, annealing at 57.5 °C for 30 s, and elongation at 72 °C for 1 min, and a final extension step at 72 °C for 5 min. A thermocycler Biorad T100, Bio-Rad Laboratories N.V., Temse, Belgium was used. Secondly, the amplified reaction products were loaded onto DNA 1000 chips (Agilent Technologies, Santa Clara, CA, USA), prepared according to the manufacturer’s recommendations. The resulting DNA fragments, which vary in length from 150 to 1500 bp, were separated by means of an Agilent 2100 Bioanalyzer (Agilent Technologies, Santa Clara, CA, USA), whose working is based on capillary electrophoresis. The 2100 Expert Software (Agilent Technologies, Santa Clara, CA, USA) was used to digitalize the ARISA fingerprints, resulting in electropherograms in ASCII formats, which were processed using the StatFingerprints package in the 2.13.0 version of the R project (The R Foundation for Statistical Computing, Vienna, Austria).

### 2.5. Shotgun Metagenomic Sequencing 

Shotgun metagenomic sequencing was chosen to obtain a snapshot of the microbial communities present at the three sites (Warsaw, Bóbrka, Białowieża) [25,26]. Shotgun metagenomic sequencing sequences give all DNA from a sample, allowing to study all the microbiota present in it [27,28]. The Illumina Nextera DNA XT kit was used for library preparation, and the 100 bp PE sequencing kit with reagent TruSeq 4000 SBS Kit v3 was used. This library preparation is based on a single tagmentation enzymatic reaction in which sample DNA is simultaneously fragmented and tagged with adapters. Then, a PCR step amplifies tagged DNA and adds sequencing indexes according to the kit indications. Samples were prepared and sequenced by Macrogen (Seoul, Korea) on the Illumina HiSeq4000 platform to generate between 5 and 10 million PE (paired-end) reads per sample. The sequencing control software used was HCS v3.3. Read quality was assessed with FastQC v0.11.5. FastQC includes multiple modules: basic statistics, per base sequence quality, per sequence quality score, per base sequence content, per sequence GC content, per base N content, sequence length distribution, sequence duplication levels, overrepresented sequences, adapter content, and kmer content. Trimmed reads (Trimmomatic v0.36) showing leading and trailing minimum qualities of 3 and minimum read length equal to 36 were submitted to the Kaiju web server for taxonomic assignments [29]. Parameters set in Kaiju included analyses of all taxonomic levels, NCBI BLAST nr+euk used as reference database, low abundance filter 0 ≤ 0.5 ≤ 10. Samples were filtered considering the lowest complexity, and mismatches were allowed in Greedy mode (Greedy max mismatches 1 ≤ 5; Greedy minimum bitscore 10 ≤ 75; Greedy max E-value 0 ≤ 0.05 ≤ 1).

### 2.6. Isolation of Culturable Epiphytic Fungi 

In order to isolate epiphytic fungi, we plated pure and 1/10 diluted leaf wash suspensions onto different types of agar solid media containing different nutrients and at different concentrations [21]. Malt Extract Agar [30,31] and Czapek dox at pH = 5 [32] were chosen because both these media are recommended for the general cultivation of fungi from a wide range of environmental niches. Furthermore, pure and 1/10 diluted leaf wash suspensions were also screened on 869 rich media plates at pH = 7 [21], 1/10 LB plates pH = 7 [33], and from minimal medium YMAb284 plates pH = 7 [21]. Plates were incubated at 30 °C. These media were chosen for their difference in nutrient content, which can favor the isolation of a wider array of culturable epiphytic fungi [30,34].

### 2.7. Improving Isolation of Culturable Epiphytic Fungi via Enrichment Cultures

Aiming to diversify the isolation of epiphytic fungi, we set up enrichment cultures where 1 mL leaf wash suspensions from Bóbrka were added to 250 mL Erlenmeyer flasks previously filled with 100 mL of Bushnell-Haas medium at pH = 7 [35] containing 0.1% (*w*/*v*) diesel-related aromatic pollutants as sole C-source, including filter-sterilized diesel, BTEX (mix of benzene, toluene, ethylbenzene, o-xylene, m-xylene, and p-xylene), polycyclic aromatic hydrocarbons—PAHs (naphthalene, fluorene, and phenanthrene), phenol, and n-hexadecane. Pollutants were directly spiked into the medium or supplemented in 2 mL Eppendorf tubes taped into the Erlenmeyer flask to allow evaporation of the volatile compounds and a saturation of the air phase inside the flask. Cultures were renovated (1:50) every three weeks, and after four transfers, aliquots were spread onto MEA and Czapek dox agar Petri dishes. All plates were incubated at 23 °C for two to four weeks. Fungi picked up from the plates were grown on MEA, Czapek dox agar, Ingestad medium [36], and a specific guaiacol lignin medium [37]. Ingestad is a minimal medium containing only macro- and micronutrients, and that was supplemented by diesel (as the only carbon source) up to 0.0025%. The guaiacol lignin medium was used to facilitate the selection of fungi that produce enzymes such as laccases and peroxidases [38].

### 2.8. Genotypic Characterization of the Isolates

Isolated fungi were cultivated in liquid MEB (malt extract broth) medium (pH = 5) at 30 °C on an orbital shaker and in 12-well plates for one week before DNA extraction. Total DNA was extracted using the PowerSoil^®^ DNA Isolation Kit (Mo Bio Laboratories Inc., Carlsbad, CA, USA) and the E.Z.N.A.^®^ Fungal DNA Mini Kit (Omega Bio-Tek, Inc., Norcross, GA, USA), according the manufacturer’s recommendations. Subsequently, a PCR reaction was performed using the primer pair ITS1F (5′-CTTGGTCATTTAGAGGTAA-3′) and ITS4R (5′- TCCTCC GCTTATTGATATGC-3′) to span the entire ITS1-5.8S-ITS2 region in the genome of the fungi [39,40]. Additionally, the primer pair TEF1-108F (5′- GAYTTCATCAAGAACATGAT-3′) and TEF1-1620R (5′- GACGTTGAADCCRACRTTGTC-3′) was also used to allow deeper taxonomic resolution of the clades *Fusarium* and *Penicillium* [41]. To perform the PCR amplification, a mastermix containing 5.5 μL FastStart 10X reaction Buffer with MgCl_2_, 1 μL dNTP mix, 2 μL of each forward and reverse Primer (0.1 mM), 0.25 μL FastStart High Fidelity polymerase (Roche Applied Science, Mannheim, Germany), 38.25 μL RNAse-free water, and 1 μL of 10^−1^ diluted template DNA (2–60 ng μL^−1^) was made for each primer pair. PCR conditions included an initial denaturation step of 2 min at 95 °C, followed by 40 cycles of denaturation at 95 °C for 30 s, annealing at 55 °C for ITS/ 50 °C for TEF1 primer, extension of the amplicon at 72 °C for 1 min, and a final extension of 10 min at 72 °C. A thermocycler Biorad T100, Bio-Rad Laboratories N.V., Temse, Belgium, was used. The length of the amplicons was checked by gel electrophoresis. DNA gel-loading dye (6X) by Thermo Fisher (US) was used before loading the samples in an agarose gel 1.5% (120 V; 45 min). Samples were sent for sequencing to Macrogen (Amsterdam, The Netherlands).

### 2.9. Laccase and Peroxidase Enzyme Assays

Three representative fungi (*Lophiostoma* sp. AT37, *F. sporotrichioides* AT11, and *P. herbarum* AT15) obtained from enrichment cultures were tested for laccase and total peroxidase enzyme assays. To test laccase and total peroxidase enzyme activities, the fungi were first grown in liquid Kimura medium (pH = 5) [42]. One plug of each fungus was added to a 100 mL Erlenmeyer containing 20 mL Kimura medium and incubated at 23 °C for two weeks. After this period, the enzyme activities were determined each 48 h using a FLUOstar^®^ Omega Plate reader (BMG LABTECH Inc., Ortenberg, Germany). The basidiomycetes *Clitocybe dealbata*, *Clitocybe nebularis* ST1, and *Ganoderma* sp. UH-M [43], which were previously characterized also for their production of laccases and peroxidases, were included as positive controls. These 3 fungi are part of UHasselt fungal collection. The laccase enzyme activity was determined in triplicate spectrophotometrically by monitoring the oxidation of 1 mmol L^−1^ 2,6-dimethoxyphenol (DMP) to 2,2′,6,6′-dimethoxydiphenoquinone in 100 mM sodium acetate buffer (pH = 5). For a final reaction sample of 200 μL, 10 μL of the culture supernatant was added to 186 μL of the sodium acetate buffer and finally, 4 μL of a 50 mM DMP was added, and the enzyme activity was immediately measured for a period of time of a minute, using a FLUOstar^®^ Omega Plate reader (BMG Labtech, Germany) set to a wavelength of 468 nm. Laccase activities (ε468 nm: 49,600 M^−1^ cm^−1^) were expressed in μU L^−1^ [44]. Enzyme units (U) are a unit of enzyme’s catalytic activity. Specifically, we present the results as micro units per liter^−1^ (μU L^−1^). The activity of nonspecific peroxidase (NsP) was determined in triplicate using 0.5 mM o-dianisidine as a substrate for oxidation in the presence of 4 mM H_2_O_2_. The activity was determined in a final reaction sample of 200 μL containing 100 μL acetate buffer (100 mM, pH = 5), 39 μL H_2_O, 26 μL of 4 mM H_2_O_2_, 10 μL of the culture supernatant, and lastly added, 25 μL of a 4 mM o-dianisidine stock (dissolved in absolute ethanol). After adding the o-dianisidine, absorbance was immediately measured for a minute at 445 nm. The peroxidase enzyme (ε445 nm: 47,665 M^−1^ cm^−1^) activities were expressed in micro units per liter medium (μU L^−1^) [45].

### 2.10. Fungal Hydroxyl Radical Production Assay 

Fungal hydroxyl radical production was estimated by determining the oxidation of the substrate terephthalate to hydroxylterephthalate [46]. Four representative fungi (*Lophiostoma* sp. AT37, *F. sporotrichioides* AT11, and *P. herbarum* AT15, and *P. citrinum* AT26), obtained from enrichment cultures, were tested for laccase and total peroxidase enzyme assays. These fungi and the positive control *Ganoderma* sp. UH-M were cultivated in 100 mL Erlenmeyers with 20 mL liquid MEB medium, in triplicate, for one week at 23 °C on an orbital shaker at 100 rpm. After one week, the mycelia were washed with Ingestad medium, which was pipetted out, and freshly prepared Ingestad medium was added to the flasks. Ingestad medium pH 5 contains 96 μM KNO_3_, 70 μM K_2_SO_4_, 63 μM KH_2_PO_4_, 58 μM K_2_HPO_4_, 732 μM NH_4_NO_3_, 36 μM Ca(NO_3_)_2_.4H_2_O, 62 μM Mg(NO_3_)_2_.6H_2_O, 13 μM HNO_3_, 5 μM H_3_BO_3_, 2 μM Mn(NO_3_)_2_.4H_2_O, 0.1 μM Zn(NO_3_)_2_.4H_2_O, 0.1 μM CuCl_2_.2H_2_O, 0.02 μM Na_2_MoO_4_.2H_2_O, and 3 μM Fe(NO_3_)_3_9H_2_O [47]. After one hour, medium was removed and 5 mL of Ingestad with glucose (0.52 g L^−1^) and 2.5 mM terephthalate were added to each Erlenmeyer flask. An amount of 100 μL of the culture supernatant was pipetted from each flask into the wells of a 96-well plate to measure absorbance at 311 nm using a FLUOstar^®^ Omega Plate reader. Measurements were done after one hour and then every day for a week. Blanks were subtracted from the measurement data, and results were expressed as area under the absorbance versus time curve (AAT).

### 2.11. MAH and PAH Tolerance Test

To perform an aromatic hydrocarbon tolerance experiment, four representative fungi (*Lophiostoma* sp. AT37, *F. sporotrichioides* AT11, *P. herbarum* AT15, and *P. citrinum* AT26) obtained from enrichment cultures were tested and two fungi were used as positive controls (*C. dealbata* and *C. nebularis* ST1). To assess the tolerance of the fungi to the aromatic hydrocarbons, fungal growth was measured regularly after exposing the fungi to naphthalene and benzene. Firstly, fungi were grown on agar plates containing Ingestad medium prepared with 2.5 g L^−1^ fructose and 2.5 g L^−1^ glucose as carbon sources. When the fungi reached a diameter of 2 cm, they were exposed to gaseous naphthalene (10 ppmV) and benzene (9000 ppmV). Concentrations of naphthalene and benzene, expressed in ppmV, were decided based on the evaluation of worldwide air pollution monitoring data related to these two compounds [48,49]. For each fungus, three replicates were set up for the control group and three replicates for the group exposed to hydrocarbons. The diameter of the fungi on the plates was measured at the start of exposure and then every two days up to 11 days.

### 2.12. Naphthalene Degradation Assay

Three representative fungi (*Lophiostoma* sp. AT37, *F. sporotrichioides* AT11, and *P. herbarum* AT15) obtained from enrichment cultures were further tested to verify degradation of the airborne pollutant naphthalene. We chose to focus on naphthalene because it is an ubiquitous airborne pollutant [50], it is the most investigated among the PAHs [51], and the naphthalene isomers are the dominant PAHs in fresh crude petroleum [52].

A static experiment in closed 500 mL Erlenmeyer flasks with solid media was performed. The selected fungi were grown on solid media in two batches, one with Kimura medium (pH = 5) and one with Ingestad medium containing 2.5 g L^−1^ fructose and 2.5 g L^−1^ glucose (pH = 5), in Erlenmeyer flasks with an additional neck for subsequent sampling with a syringe. Necks were closed with Mininert valves and screw thread (Sigma Aldrich, St. Louis, MI, USA).

When the slow- and fast-growing fungi had reached a diameter of approximately 5 to 8 cm on agar media (typically after two weeks for the fast-growing fungi), they were exposed to naphthalene (3 pg^−1^ L^−1^, expressed for convenience as 10 ppmV).

Four negative controls were included for each medium used. Additionally, controls for the unspecific absorption of the pollutants were included to evaluate the amount of pollutants that was absorbed on the mycelium of the fungi. This was investigated by spraying a CuSO_4_ solution (300 mM) on the fungi to make them metabolically inactive [53]. After two weeks of exposure to the pollutants, the naphthalene and benzene concentrations in the air phase were determined using GC-MS. Gas-tight syringes with fixed needle (PerkinElmer, Waltham, Massachusetts, USA) were used to sample air phase from the flasks. Later, GC-MS analyses were performed.

### 2.13. Analyses of Naphthalene Concentrations Using GC-MS

Volatile organic compounds (VOCs) [54] were determined using gas chromatography mass spectrometry (GC-MS). We used a 30 m capillary column DB5-MS, 0.25 mm internal diameter with a film thickness of 0.25 μm (Agilent Technologies, Santa Clara, CA, USA). The GC-MS parameters used were as follows: 1 μL injection by the TriPlus RSHautosampler (Thermo Scientific, Waltham, MA, USA), 30 s splitless mode at 280 °C, and split flow at 50 mL min^−1^. The column temperature was initially 35 °C for 1 min, then gradually increased to 245 °C at 15 °C min^−1^. The MS conditions were a scan at 33–100 in 0.2 s for benzene and 33–150 in 0.3 s for naphthalene. The GC used was a Trace 1310 gas chromatograph (Thermo Scientific, USA), and the MS was a ISQ LT Single Quadrupole Mass Spectrometer (Thermo Scientific, USA). The mass spectra of individual total ion peaks were identified by comparison with the NIST mass spectra database.

### 2.14. Statistical Analyses 

All statistical analyses were performed using the 3.2.3 version of R (The R Foundation for Statistical Computing, Vienna, Austria), and the 2.13.0 version of R was used to edit ARISA fingerprints. For multivariate datasets such as the enzyme tests, the tolerance test, and the FDA assay, ANOVA and Student’s t-test were used if the data were normally distributed and homoscedastic. Post hoc analyses for two-by-two comparisons were performed using Tukey’s honest significant differences tests. If the data were not normally distributed, log transformation of the data was done and normality was again checked using the Shapiro–Wilk test. Homoscedasticity of variances was checked using Bartlett’s test. If the data were still not normally distributed, the nonparametric Kruskal–Wallis test was performed followed by a post hoc analysis using the pairwise Wilcoxon rank sum test. Multivariate statistical techniques were applied on the ARISA fingerprints. These were analyzed using non-metric multidimensional scaling (NMDS) with the Bray–Curtis distance metric. Analyses of similarity (ANOSIM) was performed to evaluate significant differences between groups with multivariate data.

### 2.15. NCBI Accession Numbers

Raw shotgun sequencing data were submitted to the Short Read Archive of NCBI with project identifier PRJNA506726 and individual FASTQ sample IDs SRX5062447-SRX5062463.

## 3. Results 

### 3.1. Comparison of the Hornbeam Epiphytic Fungal Communities via Community Fingerprint

Community-specific profiles obtained from the hornbeam phyllosphere from the three locations (Białowieża National Park, the crude-oil polluted site of Bóbrka and the city center of Warsaw) were compared according to the above-mentioned method. A complete linkage algorithm was used to perform a cluster analysis of Bray–Curtis dissimilarity matrices inferred from the fingerprints profile. Due to the high sensitivity of the automated sequencer, the Bioanalyses software allowed us to detect between 10 and 25 peaks in the electropherograms per profile (Supplementary Figure S1). The structure of the profiles, characterized by the number and length distribution of major bands (peaks of highest relative fluorescence intensity), varied deeply between phyllosphere isolated from Białowieża National Park and that from Bóbrka.

**Figure 1:**
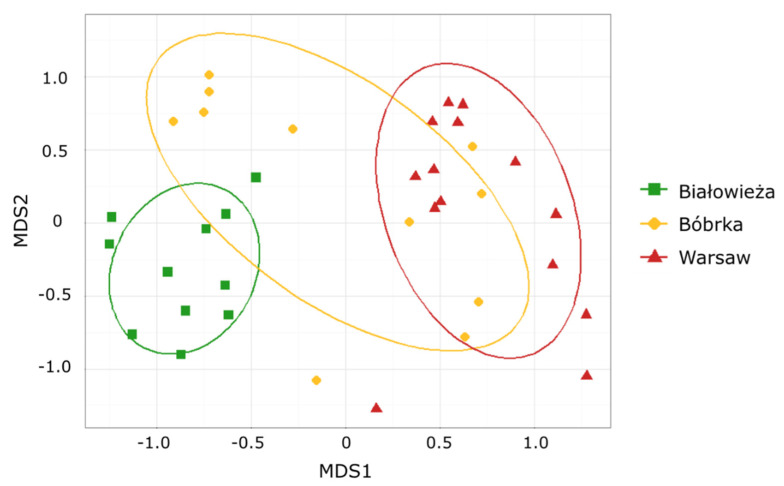
NMDS analysis of the fungal ARISA fingerprints with the Bray–Curtis distance metric (stress = 0.17) of the Białowieża National Park, the crude-oil-polluted site of Bóbrka, and the city center of Warsaw. Probability ellipses (standard deviation, *p* = 0.68) are shown for each site considered as geographically separated.

Non-metric multidimensional scaling (NMDS) analysis with the Bray–Curtis distance metric was used to analyze the fingerprints obtained from the ARISA analyses (Figure 1), with a probability ellipse (standard deviation, *p* = 0.68) for each location. The fungal communities inhabiting the phyllosphere of the hornbeam trees growing in the nonpolluted Białowieża National Park and in the polluted city center of Warsaw were significantly different (ANOSIM; R = 0.8, *p* = 0.001). Furthermore, the phyllosphere fungal communities of Białowieża and Bóbrka showed differences, although less remarkable, between their fungal communities (ANOSIM; R = 0.45, *p* = 0.001). A bigger internal variation was noticed in the fungal communities of the phyllosphere of Bóbrka in comparison to the other sampling areas. Fungal communities on each subsite within the Białowieża National Park differed less than for the other sites. Hence, in Białowieża, the lowest variation between biological replicates was observed. In addition, differences were noticeable between the phyllosphere fungi of Bóbrka and those of Warsaw; however, the separation between these communities was less strong (ANOSIM; R = 0.33, *p* = 0.001).

### 3.2. Shotgun Metagenomic Sequencing of Hornbeam Epiphytic Communities

Six samples per site (Warsaw, Bóbrka, and Białowieża) were shotgun sequenced. As described in paragraph 2.5, the read quality was assessed for all the samples, and reads were trimmed and submitted to the Kaiju web server for taxonomic assignments. A cut-off value of ≥1.0% was used at all taxonomic levels.

A mean of the sample reads for the relative abundance of fungi and bacteria at each location is provided in Table 1. From the classified reads for each site, the highest relative abundance of fungi was found in the phyllosphere of Bóbrka (calculated as percentage of 3.96%), followed by Warsaw (1.66%) and lastly, Białowieża (1.03%). The annotated phyllosphere of each site was clearly dominated by bacteria, found with percentages of 98.67, 97.86, and 95.41, respectively, in Białowieża, Warsaw, and Bóbrka.

The numbers of total sequences and of classified ones are presented in Supplementary Table S1.

**Figure 2:**
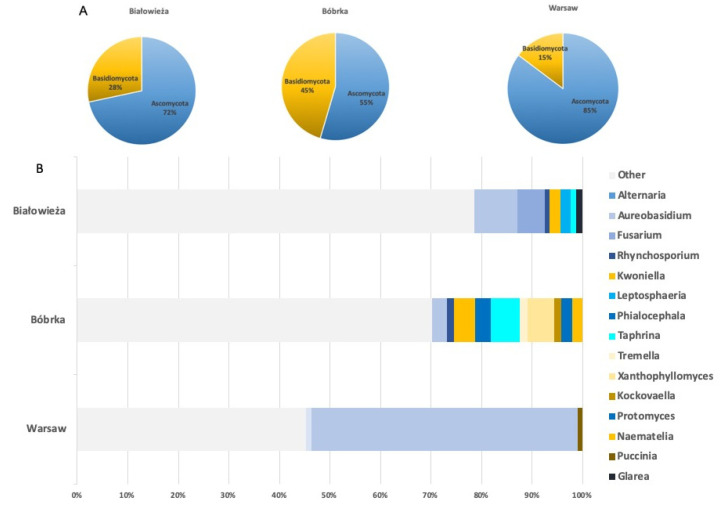
Total epiphytic fungal communities at the three locations according to shotgun metagenomic analyses. (**A**) Relative abundances for Ascomycota and Basidiomycota in the phyllosphere of the National Park of Białowieża, the crude-oil-polluted site of Bóbrka, and the city center of Warsaw. (**B**) Relative abundances of classified fungal genera, with a cut-off value of ≥1.0%.

The relative abundances of the phyla Ascomycota and Basidiomycota (Figure 2A) present on the leaves of the hornbeam trees on each site differed greatly. Warsaw had the greatest relative abundance of Ascomycota (85.16%), followed by Białowieża (71.66%), and Bóbrka had the lowest relative abundance (54.56%). Bóbrka had the highest relative abundance of epiphytic basidiomycetes (45.44%) compared to the other locations. Remarkably, no basidiomycetes, even with a lower relative abundance ≥0.1%, were detected in the phyllospheres of Białowieża and Warsaw. Even though the relative abundance of basidiomycetes in the phyllosphere of Bóbrka was high, the phyllosphere fungal communities on each site were still dominated by fungi belonging to the phylum Ascomycota.

Relative abundances of the fungal genera for each sampling site are presented in Figure 2B. A cut-off value of 1.0% was set, and all genera having an abundance greater than or equal to 1.0% are presented. The classified fungal genera, with a relative abundance ≥1.0%, belonged to the following classes: *Dothideomycetes, Sordariomycetes, Leotiomycetes, Taphrinomycetes* and *Tremellomycetes, Ascomycetes,* and *Urediniomycetes*. All these fungal classes belong to the phylum *Ascomycota,* except for *Tremellomycetes* and *Urediniomycetes,* which belong to the phylum Basidiomycota. Some fungi belonging to the class *Tremellomycetes* (*Tremella* and *Xanthophyllomyces*) were only detected in the phyllosphere of Bóbrka with a cut-off ≥1.0%.

Overall, epiphytic fungal communities were more diverse in Białowieża and Bóbrka compared to that from Warsaw. The phyllosphere from Warsaw was clearly dominated by members of the genus *Aureobasidium*, with an abundance of 52.65%. Fungi belonging to the genus *Aureobasidium* were also present, although to a lesser extent, in the phyllosphere of Białowieża (8.62%) and in Bóbrka (2.95%). *Aureobasidium* is the only genus detected at the three locations with a relative abundance ≥1.0%. The community composition and relative abundances of fungal genera also differed greatly when comparing the two sites.

### 3.3. Fungal Cultivable Isolates from the Hornbeam Leaves

Aiming to isolate and cultivate potential degraders of aromatic compounds, we decided to focus on the cultivable phyllosphere fungi isolated from the phyllosphere communities of Bóbrka and Warsaw. The 25 cultivable phyllosphere fungi (Supplementary Table S1) isolated from the crude-oil-polluted area of Bóbrka and the city center of Warsaw were all classified as Ascomycota. They belonged to the classes Dothideomycetes, Eurotiomycetes, and Sordariomycetes. Fungi belonging to the genera *Alternaria*, *Aureobasidium,* and *Fusarium*, detected in the phyllosphere after metagenome analysis, could also be cultivated. The genera *Alternaria* and *Fusarium* were detected in the metagenome of the phyllosphere of Bóbrka. The fungus *Aureobasidium pullulans* was the only species detected in all three sites, with the highest relative abundance in Warsaw hornbeam phyllosphere.

### 3.4. Laccase and Peroxidase Activities

Among the cultured epiphytic fungi, three of them were investigated for laccase and peroxidase enzyme production. *Lophiostoma* sp. AT37, *F. sporotrichioides* AT11, and *P. herbarum* AT15 were chosen considering their abundance and hydrocarbon-catabolic potential. Three fungi were used as positive controls to test the production of laccases and peroxidases: *Clitocybe dealbata*, *Clitocybe nebularis* ST1, and *Ganoderma* sp. UH-M; these are all basidiomycetes from our collection producing ligninolytic enzymes, including laccase and peroxidase.

**Figure 3:**
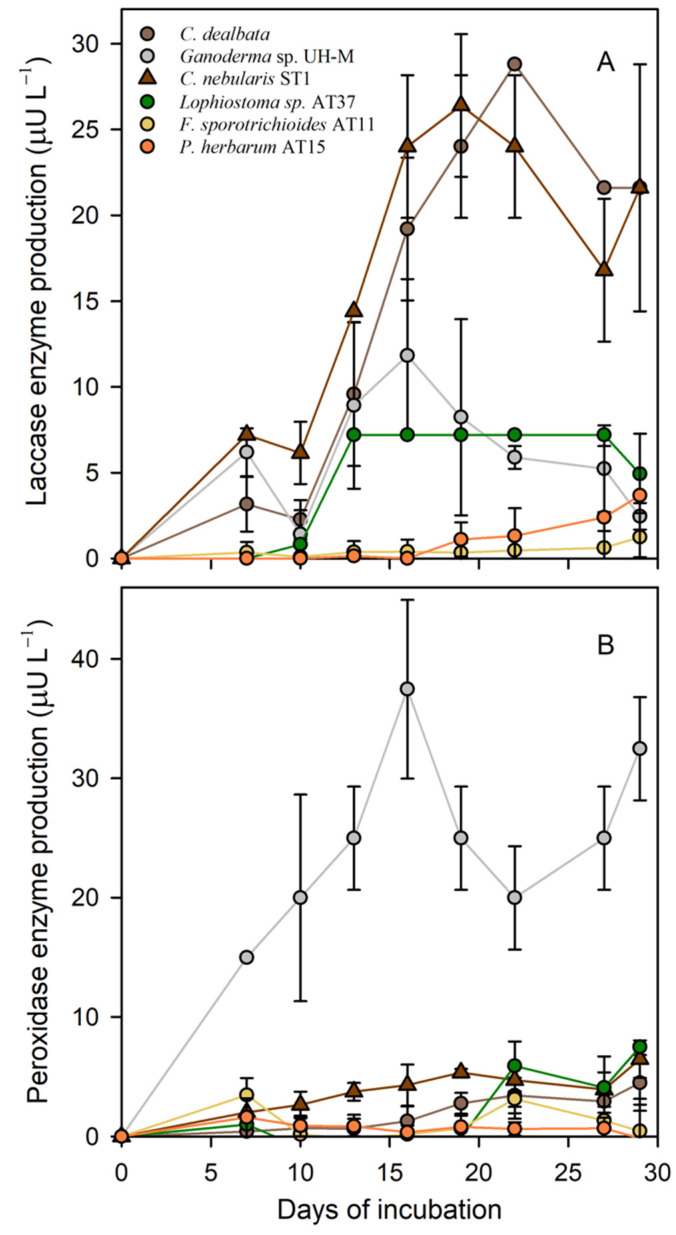
Enzyme activity. (**A**) Laccase and (**B**) peroxidase produced by time and represented as a mean of three replicates for the fungal strains *Lophiostoma* sp. AT37 (green), *F. sporotrichioides* AT11 (yellow), and *P. herbarum* AT15 (orange) and three strains used as control (grey).

Among the selected epiphytic fungal strains, *F. sporotrichioides* AT11, *P. herbarum* AT15, and *Lophiostoma* sp. AT37 produced laccase and peroxidase enzymes in Kimura medium (Figure 3). The activities of both enzymes were determined starting from day seven since the beginning of the incubation. *Lophiostoma* sp. AT37 had the comparatively highest laccase activity and started producing the enzyme from 10 days, showing an activity of 7.2 μU L^−1^ at its highest. For 14 days, the laccase activity remained steady, and after this, it decreased. *P. herbarum* AT15 and *F. sporotrichioides* AT11 started producing laccase from days 19 and 27, respectively, with activities of 3.62 μU L^−1^ and 1.24 μU L^−1^ (Figure 3). The differences in laccase activities between the different fungi were statistically not significant, but the Ascomycete *Lophiostoma* sp. AT37 together with the basidiomycetes that were used as positive controls exhibited the highest activities.

In general, the enzyme total laccases and peroxidases produced by the epiphytic fungi were comparable, though *Lophiostoma* sp. AT37 started producing laccases (day 10) earlier than peroxidases did (at 19 days). In addition, the peroxidase activity was slightly higher (7.49 μU L^−1^). *Fusarium sporotrichioides* AT11 already produced these enzymes after 7 days (3.46 μU L^−1^). However, after 10 days, no enzyme activity was detected anymore, then enzyme production resumed after 19 days. Peroxidase activities for *P. herbarum* AT15 were higher, with a peak activity of 2.28 μU L^−1^, compared to the laccase activity. For the peroxidase activity as well as for the laccase activity, the epiphytic fungus *Lophiostoma* sp. AT37 demonstrated the highest activities compared to the other epiphytic fungi.

### 3.5. Hydroxyl Radical Production

**Figure 4:**
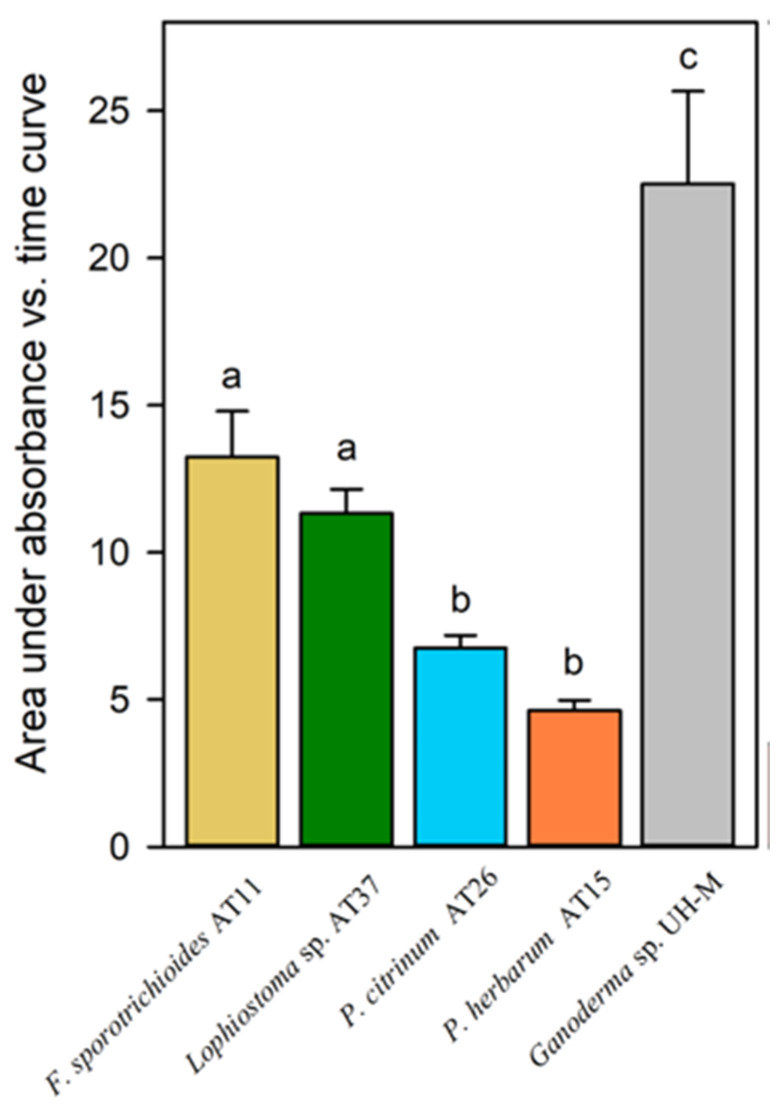
Fungal hydroxyl radical production presented as the area under the absorbance (311 nm) versus time curve. Production of hydroxyl radicals by the epiphytic fungi isolated from Bóbrka and Warsaw together with the positive control for laccase and peroxidase production *Ganoderma* sp. UH-M was measured for five days. Statistically significant differences in hydroxyl radical production between the different strains are indicated with letters above each column; columns with a different letter are significantly different (*p* < 0.05) according by ANOVA followed by post hoc test.

Hydroxyl radical production was estimated for the selected epiphytic fungi *Lophiostoma* sp. AT37, *F. sporotrichioides* AT11, *P. herbarum* AT15, and *P. citrinum* AT26. *Ganoderma* sp. UH-M was used as control (Figure 4). The hydroxyl radical production for *F. sporotrichioides* AT11 and *Lophiostoma* sp. AT37 was significantly higher than for *P. citrinum* AT26 and *P. herbarum* AT15. *Ganoderma* sp. UH-M demonstrated the significantly highest hydroxyl radical production.

### 3.6. MAH and PAH Tolerance

**Figure 5:**
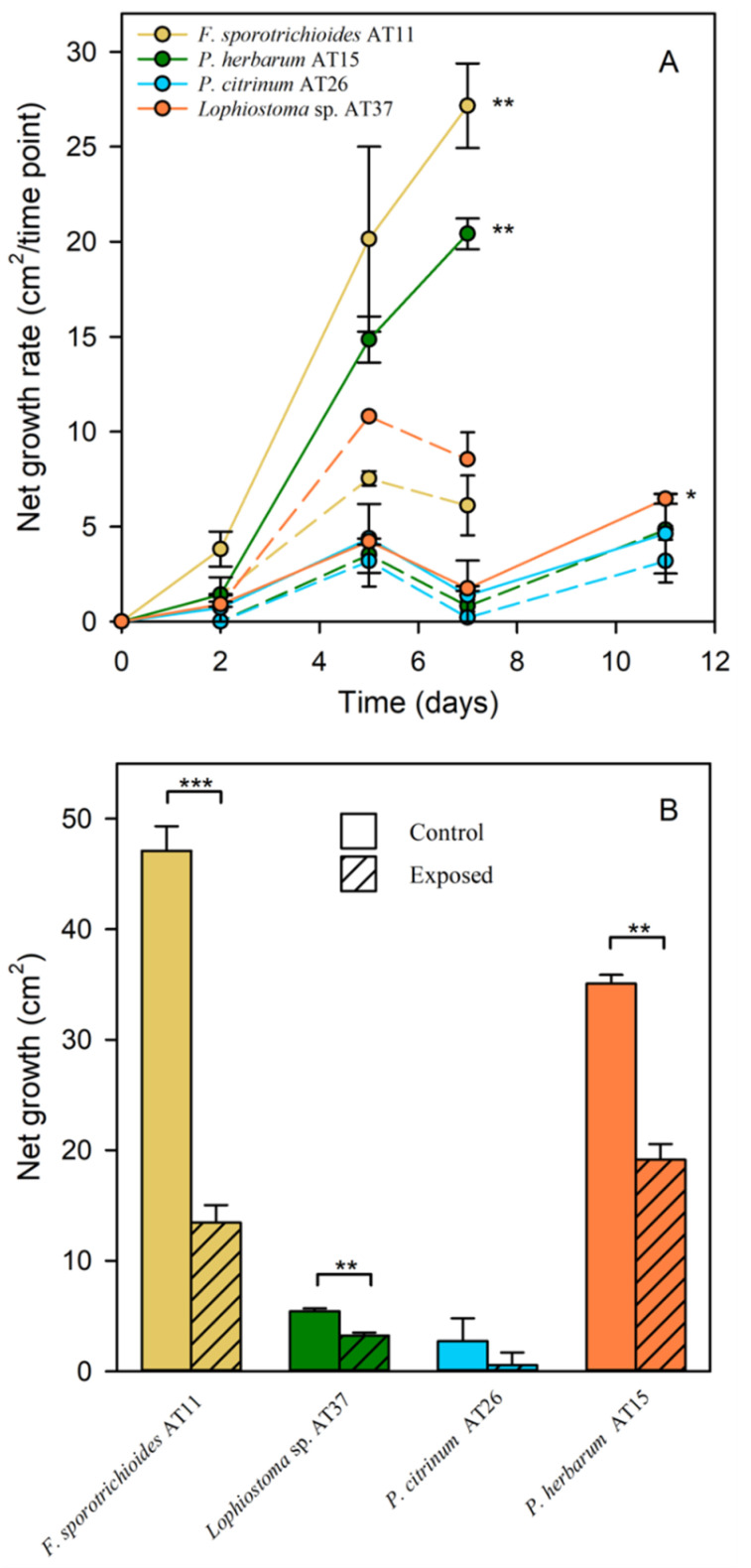
Tolerance test of epiphytic fungi, exposed to naphthalene and benzene, over time. The four selected epiphytic strains are represented (**A**) by the colored lines, showing the mean of the net growth rate per time point ± SD. Means based on the three replicates per each sample were calculated. A solid line indicates the non-exposed condition, and a dotted line indicates the exposed condition for each strain. (**B**) Net growth, at the last day of the growth assessment, for each strain. Statistically significant differences are indicated after performing Student’s t-tests by * (*p* ≤ 0.05), ** (*p* ≤ 0.01), and *** (*p* ≤ 0.001).

To assess the tolerance of the epiphytic fungi to naphthalene (10 ppmV) and benzene (9000 ppmV), an exposure experiment was performed. Fungi *Lophiostoma* sp. AT37, *F. sporotrichioides* AT11, *P. herbarum* AT15, and *P. citrinum* AT26 were investigated. The net growth rate of each fungus was determined (Figure 5A). Growth assessment was ended when the fungal mycelia reached the edge of the Petri dish. For *F. sporotrichioides* AT11 and *P. herbarum* AT15, measurements were stopped after seven days. Net growth rate of these strains was significantly lower when exposing them to the pollutants compared to the non-exposed conditions (*p* ≤ 0.01). After two days of exposure, net growth rate still increased, but for both species, a decline in growth rate was observed after five days. The net growth after seven days for *F. sporotrichioides* AT11 and *P. herbarum* AT15 is presented in Figure 5B. After this period, the net growth after exposure was significantly more inhibited for *F. sporotrichioides* AT11 (*p* ≤ 0.001) than for *P. herbarum* AT15 (*p* ≤ 0.01). *Lophiostoma* sp. AT37 and *P. citrinum* AT26 showed a lower growth compared with the others. Although, net growth, after 11 days (Figure 5B), was significantly lower for *Lophiostoma* sp. AT37 when exposed to naphthalene and benzene compared to the non-exposed condition (*p* ≤ 0.01). However, it appeared that the growth rate for this species (Figure 5A) was not as much inhibited (*p* ≤ 0.05) as it was for *F. sporotrichioides* AT11 and *P. herbarum* AT15. When comparing non-exposed and exposed conditions for *P. citrinum*, the growth was very low for all treatments.

Interestingly, the pigmentation of *F. sporotrichioides* AT11 and *P. herbarum* AT15 was altered after exposure to naphthalene and benzene. *Fusarium sporotrichioides* AT11 produced bright pink to red pigments on the Ingestad medium, but stopped producing them after exposure to the pollutants. Pigmentation of *P. herbarum* AT15 was also reduced due to exposure.

### 3.7. Naphthalene Degradation 

**Figure 6:**
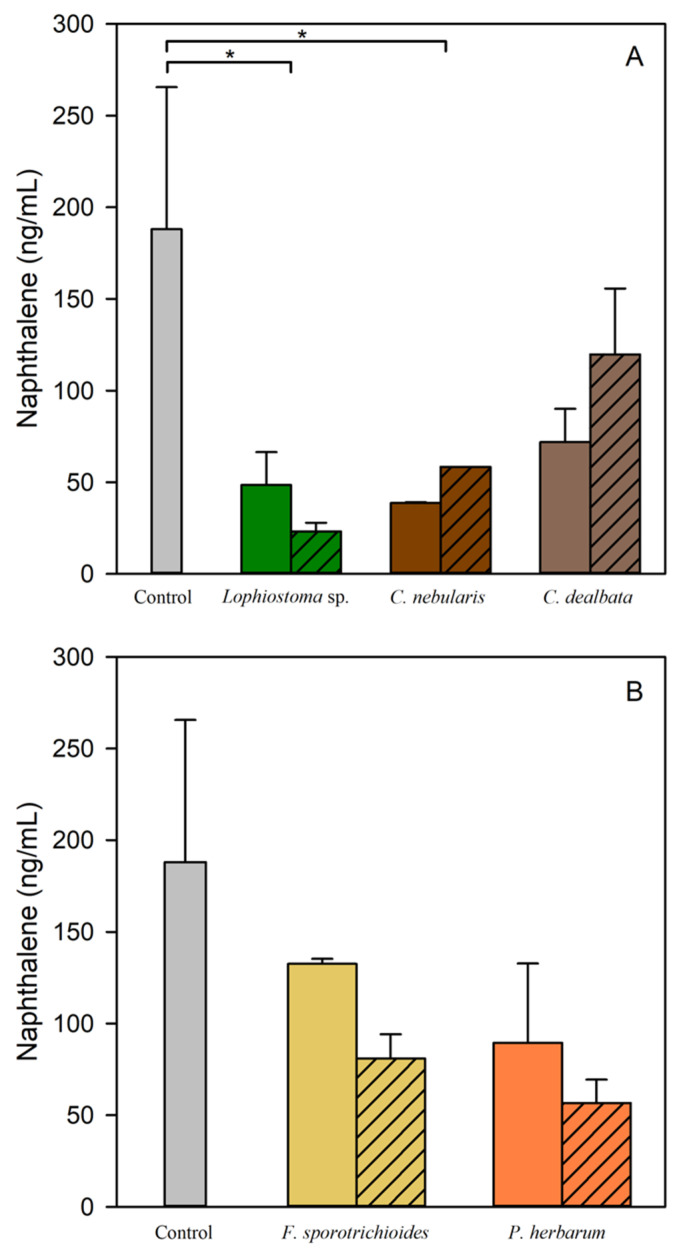
Gas chromatography mass spectrometry (GC-MS) measurement of naphthalene degradation by (**A**) the epiphytic fungus *Lophiostoma* sp. AT37 and the two strains used as positive controls for laccase and peroxidase production: *C. nebularis* and *C. dealbata*, growing on Kimura medium, and (**B**) the epiphytic fungi *F. sporotrichioides* AT11 and *P. herbarum* AT15, growing on Ingestad medium containing 2.5 g L^−1^ fructose and 2.5 g L^−1^ glucose, with shaded bars indicating the CuSO_4_-exposed condition. No-fungus media controls are indicated in gray. Statistically significant differences are indicated after performing one-way ANOVA by * (*p* ≤ 0.05).

Fungi *Lophiostoma* sp. AT37, *F. sporotrichioides* AT11, and *P. herbarum* AT15 were assessed for their naphthalene degradation potential. We observed that the airborne naphthalene concentration in the samples containing the epiphytic fungus *Lophiostoma* sp. AT37 (Figure 6A) exposed to naphthalene was significantly lower than that of the control condition (*p* ≤ 0.05). This significant reduction in naphthalene concentration was also observed in the samples containing the basidiomycete *C. nebularis* ST1 (control fungus from UHasselt collection). No significant differences in naphthalene concentration were observed for *F. sporotrichioides* AT11 and *P. herbarum* AT15. The controls containing CuSO_4_ solution were used to inactivate the fungal strains and detect naphthalene absorption on their mycelia.

## 4. Discussion

### 4.1. Hornbeam Epiphytic Fungal Communities

We investigated diversity via ARISA DNA fingerprint and we taxonomically characterized via shotgun metagenomic sequencing the fungal communities inhabiting hornbeam leaves in three different locations in Poland: the crude-oil-polluted area of Bóbrka, the city center of Warsaw, and the Białowieża National Park. Although ARISA DNA fingerprint patterns cannot reveal the taxonomic composition of the communities due to the overlapping of size classes among unrelated populations [23], some conclusions can be drawn about the diversity and distribution of the communities. We detected differences comparing fungal communities living on leaves of hornbeam trees in three different areas. Specifically, ARISA DNA fingerprinting analysis showed that epiphytic fungal communities from Białowieża differed the most from the ones in the phyllosphere of Warsaw and differed less from those in Bóbrka. According to Steven and Rebecca [55], reasons that might explain the differences in fungal community structure include genetic variation of the hosts, stand structure and size (single or clustered trees, rows, hedges) [20], properties of the soil, and nutrient availability and differences in management. However, little is known about these factors influencing microbial communities, and investigations are still ongoing. A factor that might play a role in the community differences among sites is that Bóbrka is situated in a forest area where there is a large and more diverse community of trees, whereas in the city center of Warsaw, hornbeam trees have been planted in urban parks and along roadsides. Although Bóbrka and Białowieża have different levels of pollution [20], they are basically two forest-like ecosystems and therefore with some traits more similar in comparison to a city center ecosystem. This might explain why fungal communities in Białowieża and Bóbrka are less different than when comparing Warsaw and Białowieża. Moreover, fungi in the phyllosphere of Bóbrka were probably already more adapted to the polluted conditions, more than those in Warsaw. Indeed, in Bóbrka, a massive crude oil pollution has been present since the last glacial period [19]. Kembel et al. [56] reported how the *Quercus macrocarpa* phyllosphere microbial community differs between urban and non-urban locations. They suggest that a combination of mechanisms leads to differences between urban and non-urban communities. Among those are stand isolation and size, nutrient and pollutant accumulation, plus stand management, including fertilization and litter removal.

Taking into account that it is estimated that only 5–10% of the fungal species are known in science and that the majority of species are only characterized by morphology and substrate while only less than 20% of the characterized fungal species are represented with DNA sequences in public databases [57], we performed shotgun metagenome sequencing analysis and taxonomic identification of the phyllosphere fungal communities using the software Kaiju [29]. Phylum Ascomycota was the most represented in all three locations, in accordance with Janakiev et al. [58]. We classified fungal genera with a relative abundance higher than or equal to 1.0%. The fungal phyllosphere communities of Białowieża and Bóbrka were more diverse than in Warsaw when comparing relative abundances of classified fungal genera (≥1.0%). This can also be due to the fact that Białowieża and Bóbrka are two forest-like ecosystems in comparison to the city center ecosystem. According to Laforest-Lapoint et al. [59], when anthropogenic pressure increases, urban leaf microbial communities show a reduction in the abundance. The urban environment differs strikingly from the natural forest environment mainly because the anthropogenic pressure in urban areas reduces tree fitness and longevity and/or modifies functional traits as levels of leaf macro- and micronutrients [60]. Higher temperatures can influence vegetation phenology and contribute to the heat island phenomenon [61]. Fungi belonging to the genus *Aureobasidium* were found at all three locations, and the genera *Kwoniella, Rhynchosporium,* and *Taphrina* at two locations (Białowieża and Warsaw). Jumpponen et al. [62] reported these three genera as frequently present on leaves in the fungal phyllosphere communities in temperate regions. *Aureobasidium* was described as capable of degrading mixtures of MAH and PAH [63,64], and the genus *Rhynchosporium* is known to be able to degrade aromatic polymers as lignin [65].

The most dominant colonizers of the hornbeam phyllosphere are bacteria [4]. However, when the environment is polluted by hydrocarbons, fungi are more abundant than in the nonpolluted area. This could be due to a more favorable leaf environment in terms of biochemical composition when a tree is exposed to certain concentrations of MAH and PAH. The relative abundance of bacteria is lower in the polluted areas, which might indicate that bacteria are more sensitive to air pollution [2]. Brighignal et al. [66] also mentioned that fungi are less affected or less sensitive to air pollution than bacteria are. This might explain the trend of a higher relative abundance of fungi in the phyllosphere of the polluted areas compared to a lower abundance on leaves from the National Park of Białowieża.

We decided to focus on isolating fungi from Bóbrka and Warsaw because we expected that these locations would harbor more fungi capable of tolerating and degrading AH [67]. We isolated 25 fungi from 9 genera with hydrocarbon-degrading potential. Specifically, we decided to focus on four strains: *Fusarium sporotrichioides*, *Lophiostoma* sp., *Penicillium citrinum*, and *Phoma herbarum* from the petroleum site of Bóbrka since previous studies have reported their potential as AH degraders [68,69,70,71].

### 4.2. Catabolic Enzymes Involved in the Degradation of AHs

The three epiphyte fungi investigated were reported to show low peroxidase activity but a laccase activity comparable to that of litter and wood degraders [72].

Laccases and peroxidases are among the most important enzymes known to be involved in degradation of PAHs by Basidio- and Ascomycetes. Laccases and peroxidases are excreted by fungal cells into their surroundings and have a low substrate specificity, which makes them suitable for degrading various organic pollutants, including AH [73].

The epiphytic fungus *Lophiostoma* sp. showed the highest enzyme activities for both laccase and peroxidase enzymes compared to the other isolated epiphytic fungi. *F. sporotrichioides* AT11 and *P. herbarum* AT15 were also able to produce these enzymes. However, their enzyme activities were lower, and in general, the enzyme activity of the three epiphytic fungi was lower compared to the positive control ones (*C. nebularis* ST1, *C. dealbata,* and *Ganoderma* sp. UH-M). Lower enzyme activities might be explained by the type of medium used to culture the fungi. Torres-Farrada et al. [43] used the SB-U medium with sugarcane molasses as carbon source to successfully induce ligninolytic enzyme production. 

Besides the action of ligninolytic enzymes, the production of hydroxyl radicals is another possible mechanism to degrade organic pollutants. Hydroxyl radicals are produced extracellularly by Fenton chemistry and redox cycling of quinones. They are extremely powerful oxidants [74,75]. The epiphytic fungi *F. sporotrichioides* AT11 and *Lophiostoma* sp. AT37 produced significantly more hydroxyl radicals compared to *P. citrinum* and *P. herbarum*. The Basidiomycete *Ganoderma* sp. UH-M produced the highest amount of hydroxyl radicals. This might be explained by the fact that *Ganoderma* sp. lives on the bark of trees and uses these hydroxyl radicals to degrade lignin [76]. However, in case the phyllosphere fungi producing the hydroxyl radicals on the leaves, the concentration cannot be too high otherwise the plant tissues might be subject to oxidative stress. Moreover, the production of laccase and peroxidase enzymes might damage plant tissues. Therefore, plants and their microbiome have evolved in such a way that they are able to cope with this stress [77].

### 4.3. Tolerance to MAH and PAH and Napthalene Degradation Potential

Our results indicate that the growth of the *Lophiostoma* sp. strain was inhibited by exposing it to naphthalene and benzene. However, it was more tolerant than the other epiphytic strains that were tested. *F. sporotrichioides* AT11 and *P. herbarum* AT15 were significantly more affected when exposed to naphthalene and benzene. This detrimental effect influenced the fungal pigmentation. *Fusarium sporotrichioides* AT11 produced bright pink to red pigments on the Ingestad medium, but stopped producing them after exposure to the pollutants. Pigmentation of *P. herbarum* AT15 was also reduced due to exposure. The MAH and PAH tolerance tests were performed using a nutrient-poor medium and under natural day–night cycle to simulate better the conditions these fungi are used to. When growing on nutrient-rich MEA medium and not directly exposed to sunlight, *F. sporotrichioides* AT11 did not produce these red pigments as much as on the rather nutrient-poor Ingestad medium. Pigments such as melanin (dark brown pigments), carotenoids (orange red), and lycopene (dark red) are usually produced by fungi as mechanisms of protection against environmental stress, including UV light, ionizing radiation, drought, or oxidative stress [78]. According to Pagano and Dhar [79], pigments can also be produced when less nutrients are available. The exposure of *F. sporotrichioides* AT11 to sunlight and the limited nutrients might explain the bright red pigmentation. However, in case of too high stress, it might be that the fungal metabolism and consequently the secondary metabolites production, including pigment production, are getting disturbed. This might explain the lack or reduction of pigmentation after exposure to naphthalene and benzene.

Concerning naphthalene degradation of the most promising isolates assessed via GC-MS, our results indicate different decreases of naphthalene concentrations in the air phase of samples containing *Lophiostoma* sp., *C. nebularis*, *C. dealbata*, *F. sporotrichioides,* and *P. herbarum,* which will be further verified by working with a bigger batch of samples previously cultivated in different media to assess the influence of media on naphthalene degradation [80]. Some fungi strains were reported as able to use volatile aromatic hydrocarbons as the sole source of carbon and catalyze degradation reactions [81,82]. Naphthalene degradation has been extensively studied on bacteria [83]. However, degradation pathways for naphthalene catabolism by fungi have not been deeply investigated. For instance, Hadibarata et al. [84] reported how the production of several enzymes (manganese peroxidase, lignin peroxidase, laccase, 1,2-dioxygenase, and 2,3-dioxygenase) by *Armillaria* sp. F022, a white rot fungus collected from tropical rain forest, played an important role in metabolism of naphthalene. Other phyllosphere fungi belonging to the genera *Penicillium* sp., *Aspergillus* sp., and *Thricoderma* sp. were investigated by Undugoda et al. [67] for their capability to cope with phenanthrene, naphthalene, xylene, and/or toluene as the only carbon source, and *Penicillium* sp. was the only one to tolerate and degrade all the four AHs.

## 5. Conclusions

We extended the knowledge about the epiphytic fungal microbiome on hornbeam leaves from chronically polluted areas. The epiphytic fungal communities from three different locations in Poland were compared, revealing significant differences between the city center of Warsaw and the National Park of Białowieża (nature reserve). Different factors lay behind these differences, one of them may be air pollution, which plays a role in shaping the fungal communities inhabiting the phyllosphere of hornbeam leaves. Further investigations will elucidate this aspect.

Among the isolated fungi, *Lophiostoma* sp., isolated from the phyllosphere of hornbeam in Bóbrka, revealed to be the most promising candidate to degrade airborne AH. It demonstrated the highest laccase and peroxidase activities and also produced hydroxyl radicals. Moreover, *Lophiostoma* sp. AT37 showed promising for naphthalene degradation pathways and the production of the hydroxyl radical, which can be used as a marker for the detection of fungal degradation activity in the phyllosphere. Further studies will be carried out to investigate these biological markers as indicator of degradation potential by phyllosphere fungi.

Although knowledge on fungi inhabiting the phyllosphere is still limited, this can be considered as a contribution towards the development of new bioremediation approaches to obtain cleaner air, especially supported by recent environmental policies such as, for example, the European green deal, which encourage the use of greenery and landscaping to combat indoor and outdoor air pollution.

## Figures and Tables

**Table 1:** Shotgun metagenome analysis was performed on six samples per sampling site (Białowieża, Bóbrka, and Warsaw). Values presented are expressed as means of classified fungal reads and their standard deviations (SD). Same data are presented for bacteria, which constitute the highest microbial fraction in the phyllosphere.

	Fungi		Bacteria [20]	
	Mean	SD	Mean	SD
Bóbrka	14,106.00	7097.73	340,110.50	64,229.54
Białowieża	4449.00	1011.79	426,475.33	77,314.96
Warsaw	5704.17	4734.63	336,557.33	62,557.18

## Supplementary Materials

The following are available online at https://www.mdpi.com/article/10.3390/jof7110972/s1, Table S1 Shotgun metagenome sequencing total number of reads, total number of classified fungal and bacterial reads per sample. Their means and SD are shown. Per each location (Bóbrka, Białowieża and Warsaw) six phyllosphere samples were sequenced, Table S2 Overview cultivated epiphytic fungi from Bóbrka and Warsaw, Figure S1 Gel-like image generated by ARISA bioanalyzer to analyse the fungal diversity at the three sites (Warsaw, Bóbrka and Białowieża. The first column (L) shows the reference DNA ladder. Base pair sizes are indicated next to the ladder. The lowermost (15 bp) and the uppermost (1500 bp) bands represent the markers used to align the ladder data with data from the sample wells.

## References

[1] WHO World Health Organization. https://www.who.int/news-room/detail/02-05-2018-9-out-of-10-people-worldwide-breathe-polluted-air-but-more-countries-are-taking-action.

[2] Wei X., Lyu S., Yu Y., Wang Z., Liu H., Pan D., Chen J. (2017). Phylloremediation of Air Pollutants: Exploiting the Potential of Plant Leaves and Leaf-Associated Microbes. Front. Plant. Sci..

[3] Dzierzanowski K., Popek R., Gawronska H., Saebo A., Gawronski S.W. (2011). Deposition of Particulate Matter of Different Size Fractions on Leaf Surfaces and in Waxes of Urban Forest Species. Int. J. Phytoremediat..

[4] Vorholt J.A. (2012). Microbial life in the phyllosphere. Nat. Rev. Microbiol..

[5] Weyens N., Beckers B., Schellingen K., Ceulemans R., van der Lelie D., Newman L., Taghavi S., Carleer R., Vangronsveld J. (2015). The Potential of the Ni-Resistant TCE-Degrading Pseudomonas putida W619-TCE to Reduce Phytotoxicity and Improve Phytoremediation Efficiency of Poplar Cuttings on A Ni-TCE Co-Contamination. Int. J. Phytoremediat..

[6] Sivakumar N., Sathishkumar R., Selvakumar G., Shyamkumar R., Arjunekumar K., Yadav A.N., Singh J., Rastegari A.A., Yadav N. (2020). Phyllospheric Microbiomes: Diversity, Ecological Significance, and Biotechnological Applications. Plant Microbiomes for Sustainable Agriculture.

[7] Leveau J.H. (2019). A brief from the leaf: Latest research to inform our understanding of the phyllosphere microbiome. Curr. Opin. Microbiol..

[8] Gomes T., Pereira J.A., Benhadi J., Lino-Neto T., Baptista P. (2018). Endophytic and Epiphytic Phyllosphere Fungal Communities Are Shaped by Different Environmental Factors in a Mediterranean Ecosystem. Microb. Ecol..

[9] Kirschner R. (2018). Fungi on the leaf—A contribution towards a review of phyllosphere microbiology from the mycological perspective. Ecol. Ser..

[10] Davey M., Nybakken L., Kauserud H., Ohlson M. (2009). Fungal biomass associated with the phyllosphere of bryophytes and vascular plants. Mycol. Res..

[11] Jia T., Yao Y., Guo T., Wang R., Chai B. (2020). Effects of Plant and Soil Characteristics on Phyllosphere and Rhizosphere Fungal Communities During Plant Development in a Copper Tailings Dam. Front. Microbiol..

[12] Muller T., Ruppel S. (2014). Progress in cultivation-independent phyllosphere microbiology. FEMS Microbiol. Ecol..

[13] De Silva S., Aluwihare S., Chandimala J., Jayasooriya R., Undugoda R. Trends of Floods in Sri Lanka. Proceedings of the 36th Iahr World Congress.

[14] Ghosal D., Ghosh S., Dutta T.K., Ahn Y. (2016). Current State of Knowledge in Microbial Degradation of Polycyclic Aromatic Hydrocarbons (PAHs): A Review. Front. Microbiol..

[15] Kadri T., Rouissi T., Brar S.K., Cledon M., Sarma S., Verma M. (2017). Biodegradation of polycyclic aromatic hydrocarbons (PAHs) by fungal enzymes: A review. J. Environ. Sci..

[16] Irga P.J., Pettit T.J., Torpy F.R. (2018). The phytoremediation of indoor air pollution: A review on the technology development from the potted plant through to functional green wall biofilters. Rev. Environ. Sci. Bio..

[17] Molloy S. (2006). Phenol and the phyllosphere. Nat. Rev. Microbiol..

[18] Bienkowska M., Faszcza L., Wolyniec L. (2019). Movement to Defend the Bialowieza-The Problem of the Bialowieza Forest Protection as an Example of a Values Conflict. Borderology: Cross-Disciplinary Insights from the Border Zone.

[19] Olejarz B. (2013). The Museum of the Oil and Gas Industry was owned by Ignacy Lukasiewicz in Bobrce. Przem. Chem..

[20] Imperato V., Kowalkowski L., Portillo-Estrada M., Gawronski S.W., Vangronsveld J., Thijs S. (2019). Characterisation of the Carpinus betulus L. Phyllomicrobiome in Urban and Forest Areas. Front. Microbiol..

[21] Stevens V. (2016). The Structure of Phyllospheric Microbial Communities and Their Role in Phytoremediation of Air Pollution. https://www.semanticscholar.org/paper/The-structure-of-phyllospheric-microbial-and-their-Stevens/cb5ed75d9d9c5bfad3e20d823e8ed26c35d4902a.

[22] Cardinale M., Brusetti L., Quatrini P., Borin S., Puglia A.M., Rizzi A., Zanardini E., Sorlini C., Corselli C., Daffonchio D. (2004). Comparison of different primer sets for use in automated ribosomal intergenic spacer analysis of complex bacterial communities. Appl. Environ. Microbiol..

[23] Ranjard L., Poly F., Lata J.C., Mougel C., Thioulouse J., Nazaret S. (2001). Characterization of bacterial and fungal soil communities by automated ribosomal intergenic spacer analysis fingerprints: Biological and methodological variability. Appl. Environ. Microb..

[24] Sequerra J., Marmeisse R., Valla G., Normand P., Capellano A., Moiroud A. (1997). Taxonomic position and intraspecific variability of the nodule forming Penicillium nodositatum inferred from RFLP analysis of the ribosomal intergenic spacer and Random Amplified Polymorphic DNA. Mycol. Res..

[25] Gastauer M., Vera M.P.O., de Souza K.P., Pires E.S., Alves R., Caldeira C.F., Ramos S.J., Oliveira G. (2019). A metagenomic survey of soil microbial communities along a rehabilitation chronosequence after iron ore mining. Sci. Data.

[26] Abraham B.S., Caglayan D., Carrillo N.V., Chapman M.C., Hagan C.T., Hansen S.T., Jeanty R.O., Klimczak A.A., Klingler M.J., Kutcher T.P. (2020). Shotgun metagenomic analysis of microbial communities from the Loxahatchee nature preserve in the Florida Everglades. Environ. Microbiome.

[27] Salipante S.J., Sengupta D.J., Rosenthal C., Costa G., Spangler J., Sims E.H., Jacobs M.A., Miller S.I., Hoogestraat D.R., Cookson B.T. (2013). Rapid 16S rRNA Next-Generation Sequencing of Polymicrobial Clinical Samples for Diagnosis of Complex Bacterial Infections. PLoS ONE.

[28] Tang J., Iliev I.D., Brown J., Underhill D.M., Funari V.A. (2015). Mycobiome: Approaches to analysis of intestinal fungi. J. Immunol. Methods.

[29] Menzel P., Ng K.L., Krogh A. (2016). Fast and sensitive taxonomic classification for metagenomics with Kaiju. Nat. Commun..

[30] Black W.D. (2020). A comparison of several media types and basic techniques used to assess outdoor airborne fungi in Melbourne, Australia. PLoS ONE.

[31] Wu P.-C., Su H.-J.J., Ho H.-M. (2000). A Comparison of Sampling Media for Environmental Viable Fungi Collected in a Hospital Environment. Environ. Res..

[32] Abildgren M.P., Lund F., Thrane U., Elmholt S. (1987). Czapek-Dox Agar Containing Iprodione and Dicloran as a Selective Medium for the Isolation of Fusarium Species. Lett. Appl. Microbiol..

[33] Luria S.E., Burrous J.W. (1957). Hybridization between Escherichia-Coli and Shigella. J. Bacteriol..

[34] Basu S., Bose C., Ojha N., Das N., Das J., Pal M., Khurana S. (2015). Evolution of bacterial and fungal growth media. Bioinformation.

[35] Bushnell L.D., Haas H.F. (1941). The Utilization of Certain Hydrocarbons by Microorganisms. J. Bacteriol..

[36] Ingestad T., Kahr M. (1985). Nutrition and Growth of Coniferous Seedlings at Varied Relative Nitrogen Addition Rate. Physiol. Plantarum..

[37] Thorn R.G., Reddy C.A., Harris D., Paul E.A. (1996). Isolation of saprophytic basidiomycetes from soil. Appl. Environ. Microb..

[38] Zhang W., Ren X., Lei Q., Wang L. (2021). Screening and Comparison of Lignin Degradation Microbial Consortia from Wooden Antiques. Molecules.

[39] Balseiro-Romero M., Gkorezis P., Kidd P.S., Van Hamme J., Weyens N., Monterroso C., Vangronsveld J. (2017). Characterization and degradation potential of diesel-degrading bacterial strains for application in bioremediation. Int. J. Phytoremediat..

[40] Op De Beeck M., Lievens B., Busschaert P., Declerck S., Vangronsveld J., Colpaert J.V. (2014). Comparison and validation of some ITS primer pairs useful for fungal metabarcoding studies. PLoS ONE.

[41] Orgiazzi A., Lumini E., Nilsson R.H., Girlanda M., Vizzini A., Bonfante P., Bianciotto V. (2012). Unravelling soil fungal communities from different Mediterranean land-use backgrounds. PLoS ONE.

[42] VanderMolen K.M., Raja H.A., El-Elimat T., Oberlies N.H. (2013). Evaluation of culture media for the production of secondary metabolites in a natural products screening program. Amb. Express.

[43] Torres-Farrada G., Leon A.M.M., Rineau F., Alonso L.L.L., Sanchez-Lopez M.I., Thijs S., Colpaert J., Ramos-Leal M., Guerra G., Vangronsveld J. (2017). Diversity of Ligninolytic Enzymes and Their Genes in Strains of the Genus Ganoderma: Applicable for Biodegradation of Xenobiotic Compounds?. Front. Microbiol..

[44] Teerapatsakul C., Abe N., Bucke C., Kongkathip N., Jareonkitmongkol S., Chitradon L. (2007). Novel laccases of Ganoderma sp KU-Alk4, regulated by different glucose concentration in alkaline media. World J. Microb. Biot..

[45] Claiborne A., Malinowski D.P., Fridovich I. (1979). Purification and Characterization of Hydroperoxidase-Ii of Escherichia-Coli-B. J. Biol. Chem..

[46] Page S.E., Arnold W.A., McNeill K. (2010). Terephthalate as a probe for photochemically generated hydroxyl radical. J. Environ. Monitor..

[47] Jambon I., Thijs S., Torres-Farradá G., Rineau F., Weyens N., Carleer R., Samyn P., Vangronsveld J. (2019). Fenton-Mediated Biodegradation of Chlorendic Acid—A Highly Chlorinated Organic Pollutant—By Fungi Isolated From a Polluted Site. Front. Microbiol..

[48] Jia C.R., Batterman S. (2010). A Critical Review of Naphthalene Sources and Exposures Relevant to Indoor and Outdoor Air. Int. J. Environ. Res. Public Health.

[49] Sekar A., Varghese G.K., Varma M.K.R. (2019). Analysis of benzene air quality standards, monitoring methods and concentrations in indoor and outdoor environment. Heliyon.

[50] Batterman S., Chin J.Y., Jia C., Godwin C., Parker E., Robins T., Max P., Lewis T. (2012). Sources, concentrations, and risks of naphthalene in indoor and outdoor air. Indoor Air.

[51] Abdel-Shafy H.I., Mansour M.S.M. (2016). A review on polycyclic aromatic hydrocarbons: Source, environmental impact, effect on human health and remediation. Egypt. J. Pet..

[52] Lawal A.T. (2017). Polycyclic aromatic hydrocarbons. A review. Cogent Environ. Sci..

[53] Cervantes C., Gutierrezcorona F. (1994). Copper Resistance Mechanisms in Bacteria and Fungi. FEMS Microbiol. Rev..

[54] Tisserand R., Young R., Tisserand R., Young R. (2014). 6—The respiratory system. Essential Oil Safety.

[55] Steven W.K., Rebecca C.M. (2014). Plant traits and taxonomy drive host associations in tropical phyllosphere fungal communities. Botany.

[56] Kembel S.W., O’Connor T.K., Arnold H.K., Hubbell S.P., Wright S.J., Green J.L. (2014). Relationships between phyllosphere bacterial communities and plant functional traits in a neotropical forest. Proc. Natl. Acad. Sci. USA.

[57] Hawksworth D.L. (2004). Fungal diversity and its implications for genetic resource collections. Stud. Mycol..

[58] Janakiev T., Dimkić I., Unković N., Ljaljević Grbić M., Opsenica D., Gašić U., Stanković S., Berić T. (2019). Phyllosphere Fungal Communities of Plum and Antifungal Activity of Indigenous Phenazine-Producing Pseudomonas synxantha Against Monilinia laxa. Front. Microbiol..

[59] Laforest-Lapointe I., Messier C., Kembel S.W., Brodie E.L. (2017). Tree Leaf Bacterial Community Structure and Diversity Differ along a Gradient of Urban Intensity. mSystems.

[60] Nock C.A., Paquette A., Follett M., Nowak D.J., Messier C. (2013). Effects of Urbanization on Tree Species Functional Diversity in Eastern North America. Ecosystems.

[61] Oke T.R. (1973). City size and the urban heat island. Atmos. Environ. (1967).

[62] Jumpponen A., Jones K.L., David Mattox J., Yaege C. (2010). Massively parallel 454-sequencing of fungal communities in Quercus spp. ectomycorrhizas indicates seasonal dynamics in urban and rural sites. Mol. Ecol..

[63] Dos Santos V.L., Monteiro A.D., Braga D.T., Santoro M.M. (2009). Phenol degradation by Aureobasidium pullulans FE13 isolated from industrial effluents. J. Hazard. Mater..

[64] Schoeman M.W., Dickinson D.J. (1996). Aureobasidium pullulans can utilize simple aromatic compounds as a sole source of carbon in liquid culture. Lett. Appl. Microbiol..

[65] Penselin D., Munsterkotter M., Kirsten S., Felder M., Taudien S., Platzer M., Ashelford K., Paskiewicz K.H., Harrison R.J., Hughes D.J. (2016). Comparative genomics to explore phylogenetic relationship, cryptic sexual potential and host specificity of Rhynchosporium species on grasses. BMC Genom..

[66] Brighigna L., Gori A., Gonnelli S., Favilli F. (2000). The influence of air pollution on the phyllosphere microflora composition of Tillandsia leaves (Bromeliaceae). Rev. Biol. Trop..

[67] Ljs U., Kannangara S., Sirisena D. (2016). Aromatic Hydrocarbon Degrading Fungi Inhabiting the Phyllosphere of Ornamental Plants on Roadsides of Urban Areas in Sri Lanka. J. Bioremediat. Biodegrad..

[68] Hong J.W., Park J.Y., Gadd G.M. (2010). Pyrene degradation and copper and zinc uptake by Fusarium solani and Hypocrea lixii isolated from petrol station soil. J. Appl. Microbiol..

[69] Kirk P.W., Dyer B.J., Noé J. (1991). Hydrocarbon Utilization by Higher Marine Fungi from Diverse Habitats and Localities. Mycologia.

[70] Barnes N.M., Khodse V.B., Lotlikar N.P., Meena R.M., Damare S.R. (2018). Bioremediation potential of hydrocarbon-utilizing fungi from select marine niches of India. 3 Biotech..

[71] Hughes K.A., Bridge P., Clark M.S. (2007). Tolerance of Antarctic soil fungi to hydrocarbons. Sci. Total Environ..

[72] Janusz G., Pawlik A., Sulej J., Swiderska-Burek U., Jarosz-Wilkolazka A., Paszczynski A. (2017). Lignin degradation: Microorganisms, enzymes involved, genomes analysis and evolution. FEMS Microbiol. Rev..

[73] Tuomela M., Hatakka A. (2011). Oxidative Fungal Enzymes for Bioremediation. Compr. Biotechnol..

[74] Krueger M.C., Bergmann M., Schlosser D. (2016). Widespread ability of fungi to drive quinone redox cycling for biodegradation. FEMS Microbiol. Lett..

[75] Rineau F., Roth D., Shah F., Smits M., Johansson T., Canback B., Olsen P.B., Persson P., Grell M.N., Lindquist E. (2012). The ectomycorrhizal fungus Paxillus involutus converts organic matter in plant litter using a trimmed brown-rot mechanism involving Fenton chemistry. Environ. Microbiol..

[76] De Menezes T.A., Bispo A.S., Koblitz M.G., Vandenberghe L.P., Kamida H.M., Goes-Neto A. (2016). Production of Basidiomata and Ligninolytic Enzymes by the Lingzhi or Reishi Medicinal Mushroom, Ganoderma lucidum (Agaricomycetes), in Licuri (Syagrus coronata) Wastes in Brazil. Int. J. Med. Mushrooms.

[77] Weyens N., Thijs S., Popek R., Witters N., Przybysz A., Espenshade J., Gawronska H., Vangronsveld J., Gawronski S.W. (2015). The Role of Plant-Microbe Interactions and Their Exploitation for Phytoremediation of Air Pollutants. Int. J. Mol. Sci..

[78] Lysenko S.V., Liakh S.P. (1977). The protective role of pigments against UV rays in fungi isolated from the mesosphere. Mikrobiologiia.

[79] Pagano M.C., Dhar P.P. (2015). Fungal pigments: An overview. Fungal Biomolecules: Sources, Applications and Recent Developments.

[80] Dsouza G.C., Sheriff R.S., Ullanat V., Shrikrishna A., Joshi A.V., Hiremath L., Entoori K. (2021). Fungal biodegradation of low-density polyethylene using consortium of Aspergillus species under controlled conditions. Heliyon.

[81] Kennes C., Veiga M.C. (2004). Fungal biocatalysts in the biofiltration of VOC-polluted air. J. Biotechnol..

[82] Jin Y., Veiga M., Kennes C. (2006). Performance optimization of the fungal biodegradation of pinene in gas-phase biofilter. Process. Biochem..

[83] Zeinali M., Vossoughi M., Ardestani S.K. (2008). Naphthalene metabolism in Nocardia otitidiscaviarum strain TSH1, a moderately thermophilic microorganism. Chemosphere.

[84] Hadibarata T., Yusoff A.R.M., Aris A., Kristanti R.A. (2012). Identification of naphthalene metabolism by white rot fungus *Armillaria* sp. F022. J. Environ. Sci..

