# Supplementary material for: Exploring the Diversity and Aromatic Hydrocarbon Degrading Potential of Epiphytic Fungi on Hornbeams from Chronically Polluted Areas
Source: J Fungi (Basel). 2021 Nov 16;7(11):972. doi: 10.3390/jof7110972 (PMC8620586; doi:10.3390/jof7110972)
Supplement: Supplementary file 1 [file jof-07-00972-s001.zip › jof-1399951-supplementary.pdf]

Supplementary material

Supplementary Table 1 Shotgun metagenome sequencing total number of reads, total number of classified fungal and bacterial reads per sample. Their means and SD are shown. Per each location (Bóbrka, Białowieża and Warsaw) six phyllosphere samples were sequenced.

	**Total number of reads**	**Total number of classified fungal reads**	**Total number of classified bacterial reads**	**Fungi**		**Bacteria**	
				mean	SD	mean	SD
**Bóbrka 1**	305,400	16,733	285,818	14,106.00	7,097.73	340,110.50	64,229.54
**Bóbrka 2**	413,132	9,075	400,744				
**Bóbrka 3**	442,307	7,896	42,854				
**Bóbrka 4**	330,779	21,241	304,252				
**Bóbrka 5**	297,404	21,870	271,924				
**Bóbrka 6**	349,848	6,809	339,359				
**Białowieża 1**	485,062	5,536	475,564	4,449.00	1,011.79	426,475.33	77,314.96
**Białowieża 2**	465,702	4,254	458,499				
**Białowieża 3**	493,303	2,903	487,623				
**Białowieża 4**	481,196	4,468	473,548				
**Białowieża 5**	335,726	4,478	328,274				
**Białowieża 6**	332,377	4,160	322,688				
**Warsaw 1**	419,482	12,590	266,920	5,704.17	4,734.63	336,557.33	62,557.18
**Warsaw 2**	386,176	11,254	404,347				
**Warsaw 3**	259,786	3,409	381,093				
**Warsaw 4**	374,014	4,332	251,606				
**Warsaw 5**	351,473	10,837	366,668				
**Warsaw 6**	272,565	8,245	266,920				

Supplementary Table 2. Overview cultivated epiphytic fungi from Bóbrka and Warsaw.

Site	Class	Genus	Species	Strains
**Bóbrka**	Dothideomycetes	*Alternaria*	*alternata*	AT37; AT11; AT14; AT28
	Dothideomycetes	*Cladosporium*	sp.	AT23; AT33
	Sordariomycetes	*Fusarium*	*lateritium*	AT6
	Sordariomycetes	*Fusarium*	*sporotrichiodes*	AT8; AT11; AT35
	Sordariomycetes	*Fusarium*	*avenaceum*	AT5; AT17; AT18; AT21
	Sordariomycetes	*Fusarium*	*proliferatum*	AT22
	Dothideomycetes	*Lophiostoma*	sp.	AT37
	Eurotiomycetes	*Penicillium*	*citrinum*	AT10
	Dothideomycetes	*Phoma*	*herbarum*	AT15; AT16
	Sordariomycetes	*Sarocladium*	*strictum*	AT4
**Warsaw**	Sordariomycetes	*Acremonium*	*frucatum*	AT3
	Dothideomycetes	*Aureobasidium*	*pullulans*	AT2; AT20; AT25
	Dothideomycetes	*Cladosporium*	sp.	AT1

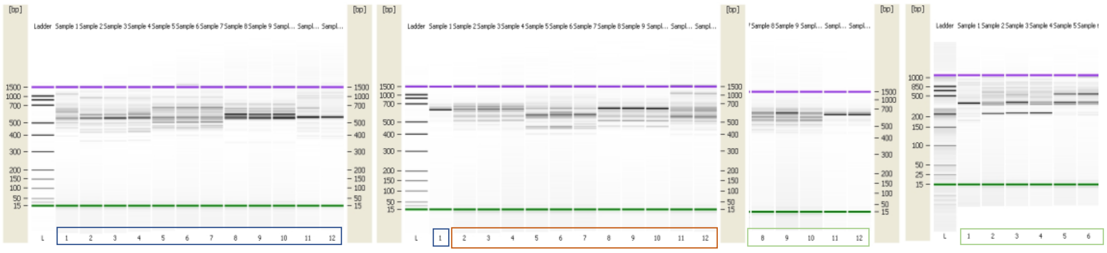

Supplementary Figure 1. Gel-like image generated by ARISA bioanalyzer to analyse the fungal diversity at the three sites (Warsaw, Bóbrka and Białowieża. The first column (L) shows the reference DNA ladder. Base pair sizes are indicated next to the ladder. The lowermost (15 bp) and the uppermost (1500 bp) bands represent the markers used to align the ladder data with data from the sample wells.
